# Concerted evolution of metabolic rate, economics of mating, ecology, and pace of life across seed beetles

**DOI:** 10.1073/pnas.2205564119

**Published:** 2022-08-09

**Authors:** Göran Arnqvist, Johanna Rönn, Christopher Watson, Julieta Goenaga, Elina Immonen

**Affiliations:** ^a^Animal Ecology, Department of Ecology and Genetics, Evolutionary Biology Center (EBC), Uppsala University, SE75236 Uppsala, Sweden;; ^b^Evolutionary Biology, Department of Ecology and Genetics, EBC, Uppsala University, SE75236 Uppsala, Sweden

**Keywords:** metabolic rate, life history evolution, sexual selection, sexually antagonistic coevolution, mating system

## Abstract

Male–female coevolution has taken different paths among closely related species, but our understanding of the factors that govern its direction is limited. While it is clear that ecological factors, life history, and the economics of reproduction are connected, the divergent links are often obscure. We propose that a complete understanding requires the conceptual integration of metabolic phenotypes. Metabolic rate, a nexus of life history evolution, is constrained by ecological factors and may exert important direct and indirect effects on the evolution of sexual dimorphism. We performed standardized experiments in 12 seed beetle species to gain a rich set of sex-specific measures of metabolic phenotypes, life history traits, and the economics of mating and analyzed our multivariate data using phylogenetic comparative methods. Resting metabolic rate (RMR) showed extensive evolution and evolved more rapidly in males than in females. The evolution of RMR was tightly coupled with a suite of life history traits, describing a pace-of-life syndrome (POLS), with indirect effects on the economics of mating. As predicted, high resource competition was associated with a low RMR and a slow POLS. The cost of mating showed sexually antagonistic coevolution, a hallmark of sexual conflict. The sex-specific costs and benefits of mating were predictably related to ecology, primarily through the evolution of male ejaculate size. Overall, our results support the tenet that resource competition affects metabolic processes that, in turn, have predictable effects on both life history evolution and reproduction, such that ecology shows both direct and indirect effects on male–female coevolution.

## Significance

Coevolution between females and males has led to remarkable differences between the sexes but has taken very different routes, even in closely related animal species, for reasons that are not well understood. We studied the physiological processes that convert resources into offspring (metabolism) in males and females of several related beetle species. We found that ecological factors dictate metabolic rate, which, in turn, have predictable direct and indirect effects on male–female coevolution. Our findings suggest that a complete understanding of differences between the sexes requires an understanding of how ecology affects metabolic processes and how these differ in the sexes.

Females and males are interdependent due to the necessity of reproduction, and male–female coevolution is responsible for a tremendous diversity of sexually dimorphic traits across animals. In most groups, however, even closely related species vary strikingly in the form and degree of elaboration of sexually dimorphism characters because male–female coevolution has taken different routes in different species. The classic explanation for such differences is that ecology dictates the economics of reproduction, which, in turn, shapes mating systems and sexual selection (1). Sexual dimorphism indeed correlates with ecological factors in a wide range of taxa [e.g., (2–5)]. To complicate matters, however, theory suggests that life histories and mating systems should be intimately linked (6, 7), and several studies have documented covariation across species between life history traits, most commonly body size [e.g., (2, 4, 8)], and mating systems or indices of sexual selection. However, the causal links between environmental factors, life history, and sexual selection are typically more or less obscure. Groups where mating system evolution and male–female coevolution involves sexual conflict (9) can serve as an example of this, since ecology is expected to play a particularly central role here because the direct costs and benefits of male–female interactions are typically contingent upon both extant ecological conditions (10–12) and life histories (13). Here, linking ecology with the economics of mating, sexual conflict, and sexual dimorphism has proven very difficult indeed (14, 15). Thus, while it is clear that ecology, life history, and reproduction should form a complex and interrelated trinity showing concerted evolution, our understanding of this multivariate coevolution is limited.

**Fig. 1.**
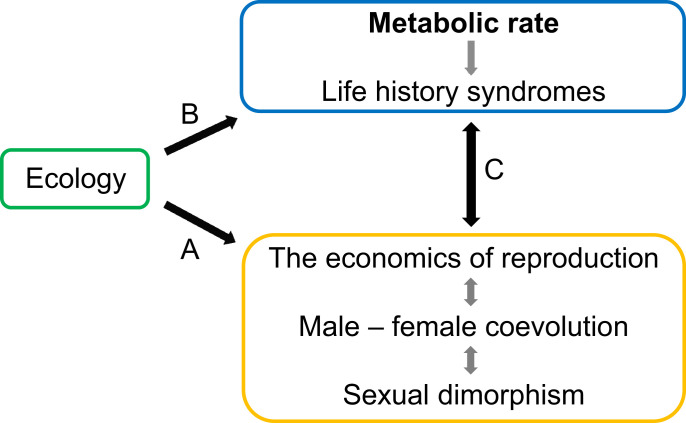
Key links between ecology, life history and reproduction. (*A*) Relationships between male–female coevolution and ecological conditions across taxa are thought to arise through direct ecological effects on the economics of reproduction, although such links are rarely well understood. However, ecology may also have important indirect effects on male–female coevolution. (*B*) In particular, resource availability is predicted to have major general effects on the evolution of metabolic rate, which in turn should push life history evolution along a physiologically constrained path of variation (i.e., the pace of life). (*C*) Such evolution of metabolic rate and life histories is predicted to have profound effects on male–female coevolution and sexual dimorphism.

We suggest that a more complete understanding of the evolutionary diversification of sexually dimorphic traits requires the conceptual integration of metabolic phenotypes into the above-mentioned trinity (Fig. 1). It has recently become clear that metabolic rate plays an important role in both ecology (16) and life history evolution (17). Suites of life history traits often differ markedly between species and tend to fall along a slow–fast pace-of-life (POLS) continuum across divergent taxa (17–19). Species on the fast end of this continuum are predicted to show relatively high metabolic rate, small body size, short life span, and early reproductive maturity, echoing classic ideas of a role for r- versus K-selection in life history evolution (20, 21). Ecological factors clearly have a central role in POLS evolution (19), as evidenced by the fact that environmental conditions and life histories often covary across species across divergent groups (18). Species experiencing more frequent or severe resource limitation tend to show a slower pace of life compared to species living in environments where resource competition is less intense. This concerted evolution of multiple traits is believed to arise from correlational selection involving underlying physiological traits (17), where metabolic rate constitutes the nexus (16). Here, lower resource availability is believed to select for lower metabolic rate (22), which, in turn, pushes life history evolution along a physiologically constrained path of variation toward slower life histories (17, 23).

Indirect ecological effects on male–female coevolution may result from the evolution of metabolic rate in several predictable ways. For example, low resource availability and a slower POLS will result in increased polyandry and thus increased sperm competition (24). In addition, ecological effects on metabolic rate and POLS may be sex specific (25, 26). Males are generally predicted to have higher optimal POLS than females (27, 28), and males tend to show a faster POLS than females (29). Further, sexual dimorphism in POLS is related to mating system in several groups (30). One possibility for this observation is that this ultimately involves sex-specific evolution of metabolic rate. Unfortunately, there are few direct studies of sex-specific selection on metabolic rate, but they do suggest that selection on metabolic rate differs between the sexes (31–34). If selection is stronger in males, then increased resource availability could generate a more rapid evolution of metabolic rate in males. This would then have cascading effects on sexual dimorphism in life histories and on male–female coevolution. This vital association has, to our knowledge, never been tested.

To some extent, our ability to gain detailed insight into this coevolutionary trinity (Fig. 1) is constrained by the fact that key phenotypes are best quantified under standardized common garden conditions (35), which is not feasible in many taxa. Because metabolic rate and life history traits are both affected by environmental conditions, this may blur or even confound underlying patterns of correlated evolution between traits (35, 36), representing a general problem in phylogenetic comparative studies: For traits that are sensitive to direct environmental impact, separating shared environmental effects from shared ancestry is challenging (35, 37, 38). Further aggravating the situation, some key phenotypes require controlled common garden experiments for their very quantification. This applies to traits such as phenotypic plasticity (39) and tolerance to environmental stress (40). In the current context, a complete understanding of sexual selection and male–female coevolution and how this relates to metabolic rate, ecology, and life histories requires quantitative comparative data on the economics of mating interactions (i.e., costs and benefits); however, such data are scarce indeed (14, 41, 42) as they require standardized experiments. When the trinity discussed above involves potentially sexually antagonistic traits or the evolution of mating effort, experimental data on the economics of mating is imperative (9, 43, 44), but currently very scarce (15, 41, 42).

In this paper, we combined a large set of common garden experiments with phylogenetic comparative approaches to test for a central role of metabolic rate in the sex-specific coevolutionary dynamics of ecology, life history (11 traits), and the economics of mating (17 traits) across 12 species of seed beetles (Bruchinae), a cosmopolitan subfamily of leaf beetles. Our primary goals were to test the four specific predictions i) that the evolution of metabolic rate has a central role in life history evolution, ii) that ecology is predictably linked to the main coevolutionary trajectory of metabolic rate and life history variation, iii) that this axis of variation shows specific links with evolution of the economics of reproduction and male–female coevolution, and finally iv) that the evolution of metabolic rate shows accelerated evolution in the sex with the highest optimal metabolic rate, which is males in seed beetles. These predictions are detailed below. Seed beetles are particularly well suited for this endeavor, as they are amenable for experimental studies but also because they are an important model system in ecology and evolution (45, 46); a very large number of studies of their ecology, life history, and reproduction collectively form the empirical basis for the predictions made here.

1. We predict that higher metabolic rate should show correlated evolution with a life history vector describing smaller body size, earlier reproductive maturity, and shorter life span. This prediction is well-founded in theory, but clear phylogenetically controlled evidence is not abundant (35, 36).
2. Seed beetles (Bruchinae) are granivorous, and the majority of the ca. 1,650 species are host specific and feed on the seeds of legume (Fabaceae) host plants during larval development, from which they emerge as adults (45, 47, 48). The host legumes are either annual herbs or perennial trees, and this dichotomy has major ramifications for seed beetle ecology (47, 49). When herbaceous host plants set seed, this provides a seasonal and ephemeral burst of resources for seed beetles. Because this cannot sustain a high seed beetle population throughout the year, seed beetles are functionally univoltine, and population sizes and seed infestations rates are both relatively low in the field. In contrast, perennial trees provide resources throughout the year as retained seed pods on the tree and/or on the ground, enabling polyvoltine seed beetle populations to build up high and more stable local population densities. The proportion of seeds infested by beetles is a direct measure of realized resource competition: While seed infestation rates of annual herbs are typically a few percent or less, perennial trees often show infestation rates of more than 75% (47, 49), sometimes even reaching over 95% (50–52), and legume trees suffer more from seed predation than do herbs in the wild (53). Because of elevated resource competition, we therefore predict that the utilization of trees as hosts should show correlated evolution with a lower metabolic rate and, consequently, a slower POLS across the sexes.
3. Seed beetle mating systems show very pronounced variation across species (54, 55), ranging from scramble competition with conventional sex roles and female mate choice (56–58) to sex-role reversal dominated by male mate choice, where females search for and actively court males (59–61). Previous work has shown that the economics of mating differs considerably across species, suggesting that male ejaculate is a key source for this variation. Last male sperm precedence is high (62, 63), and males thus benefit from matings in terms of fertilizations; however, matings also carry significant energetic costs for males (60, 64, 65). To females, apart from providing sperm, mating carries multiple direct costs and benefits. Among the former are internal injury and scarring caused by spiny male genitalia (66–69) and the receipt of specific toxic ejaculate substances (70, 71). However, large ejaculates carry substantial benefits to females through the provisioning of water (72, 73) and nutrients (74–76). The balance of these costs and benefits varies across species (77), such that remating carries net costs to females of some species (78) but sizeable net benefits to others (76). Higher and more stable local population densities should not only be associated with elevated resource competition but also with elevated reproductive competition. In line with theory, experimental evolution under high reproductive competition results in the evolution of larger male ejaculate size and reduced cost of mating to females in seed beetles (79, 80). We thus predict that the utilization of perennial trees as hosts should be associated with large male ejaculates, a resulting higher cost of mating in males and a lower cost of mating in females, since females benefit from large ejaculates. This association would be strengthened by a relatively higher male allocation to mate finding and thus a lower allocation to ejaculate size under conditions of low and more variable population densities (81). The above predicts an association between POLS and the economics of mating, which is indirect, being caused by a shared dependency with ecology. However, POLS should also directly affect the economics of mating in a manner aligned with the above prediction: A slower POLS will be associated with higher lifetime polyandry in females, which will result in increased sperm competition, in turn generating selection for higher mating effort and ejaculate size in males in this system (24).
4. Because optimal POLS is predicted to be higher in males than in females in seed beetles (81–83), a release from metabolic constraints brought about by increased resource availability should generate a more rapid evolution in male metabolic rate than in female metabolic rate. We thus predict a slope steeper than one in which male metabolic rate is regressed on female metabolic rate across species.

## Results

### Experimental Results.

The results of our experiment on metabolic phenotypes was analyzed in a full repeated measures ANOVA of resting metabolic rate (RMR), using species, sex, and mating status (virgin or mated) as between-subjects factors and body size as a between-subjects covariate. Here, the four cycles represent four repeated measures and were treated as a within-subjects effect. The results of this model (*SI Appendix*, Table S1) showed very marked and significant overall differences between species, sexes, and mating status. Body size showed a strong positive overall relationship with RMR, and males generally had a higher RMR than females, although sexual dimorphism in RMR varied markedly and significantly across species. Further, the metabolic response to mating differed significantly across species and sexes. In fact, RMR was higher in mated than in virgin individuals in about half of all species and lower in about half, and this was true in both sexes (*SI Appendix*, Dataset S1). An analogous model of variation in respiratory quotient (RQ) revealed much more subtle, but still significant, effects (*SI Appendix*, Table S2). Females showed a somewhat higher RQ than males overall, and mated individuals showed a higher RQ than virgins, although both of these effects varied significantly in magnitude across species.

To analyze variation in the cost of mating interactions to males across species, we fitted a linear model of male life span, including species and treatment (virgin or mated) as factors and male body weight (standardized within species) as a covariate, as well as all four interaction terms. Body weight had an overall positive effect on male life span (*F*_1,408_ = 13.7, *P* < 0.001), but none of the interactions involving body size were significant. Species differed in life span (*F*_11,408_ = 424.1, *P* < 0.001), virgin males lived considerably longer (*F*_1,408_ = 521.4, *P* < 0.001), and most importantly, species differed markedly with regards to how much life span was reduced as a result of mating interactions (*F*_11,408_ = 303.6, *P* < 0.001): Mating reduced male life span the most in *Megabruchidius dorsalis* (by 46%) and least in *Bruchidius dichrostachydis* (by 15%).

Variation in the economics of mating and reproduction in females across species was analyzed in a linear model of female life span including species, treatment (see *Materials and Methods*), and their interaction as factors and female body weight (standardized within species) as a covariate. Body weight had a strong overall positive effect on female life span (*F*_1,734_ = 85.0, *P* < 0.001). Species differed in female life span (*F*_11,734_ = 475.3, *P* < 0.001), and treatment had very large effects on life span (*F*_3,734_ = 252.6, *P* < 0.001), where females from treatment groups A and B lived longer overall than females from B and C. However, treatment effects differed markedly across species (*F*_33,734_ = 7.7, *P* < 0.001), reflecting differences in cost of mating, multiple mating, and reproduction to females across species. We note that although mating per se imposed a significant overall life span reduction in females (marginal mean life span, 22.58 d in A [virgins] and 21.27 d in B [mated]; Tukey’s honestly significant difference test: *P* < 0.001), the strength and even sign of this effect varied considerably across species; while seven species showed a life span reduction, five species showed a life span extension (i.e., a net benefit) as a result of mating (*SI Appendix*, Dataset S1).

Ejaculate size varied greatly across species (*F*_11,46_= 35.7, *P* < 0.001), with the ejaculate in *M. dorsalis* being 129 times the size of the ejaculate in *Callosobruchus chinensis*(*SI Appendix*, Dataset S1). The postmating ejaculate processing rate inside females also differed dramatically between species. It was lowest in *Callosobruchus phaseoli* (*b* = 0.003; 95% CI: −0.00205 to 0.00832) and highest in *Amblycerus robiniae* (*b* = 0.374; 95% CI: 0.16091 to 0.58793) (*SI Appendix*, Fig. S4). This corresponds to the time point at which 50% of the ejaculate is depleted, being more than 9 d for the former species and less than 2 h for the latter. Yet, we note that ejaculate processing rate was not significantly correlated with any other trait across species (*SI Appendix*, Datasets S2–S4).

### Phylogenetic Signal.

The overall phylogenetic signal was modest across traits (*SI Appendix*, Table S3), which should be seen in light of the fact that the deepest bifurcation in the phylogeny used here dates to about 50 Mya (84). Mean Pagel’s λ was 0.51 (range: 0.0 to 0.99), with 7 out of 28 traits showing significant phylogenetic signal. Mean Blomberg’s K was 0.59 (range: 0.27 to 1.29), with only 4 out of 28 traits showing any significant signal. Signal was highest for ejaculate volume and cost of mating in males and for RQ in both sexes.

### Pattern of Covariation within Life History and Reproductive Variables.

Our data include 11 life history traits and 17 reproductive traits measured in 12 species, and many correlations between pairs of variables were sizeable (*SI Appendix*, Datasets S2–S4). Because of the inferential difficulties that follow from the multidimensional nature of our data, we used a multivariate analytical approach. We first characterized the multivariate pattern of correlated evolution within the two main trait sets by principal component analyses using both species data (phylogenetically uncontrolled) and phylogenetic independent contrasts (PICs; controlling for shared ancestry). To enable hypothesis testing, we followed the inferential rationale of Viera (85) as implemented in PCAtest v.0.0.1 (86) in R v.4.1.2 (87). This route employs randomization tests (here, 10,000 iterations), which allowed us to i) assess the statistical significance of PCs (i.e., test for nonrandom structure among the variables) and ii) test the contributions of individual variables to the major axes of variation (i.e., the loadings).

**Fig. 2.**
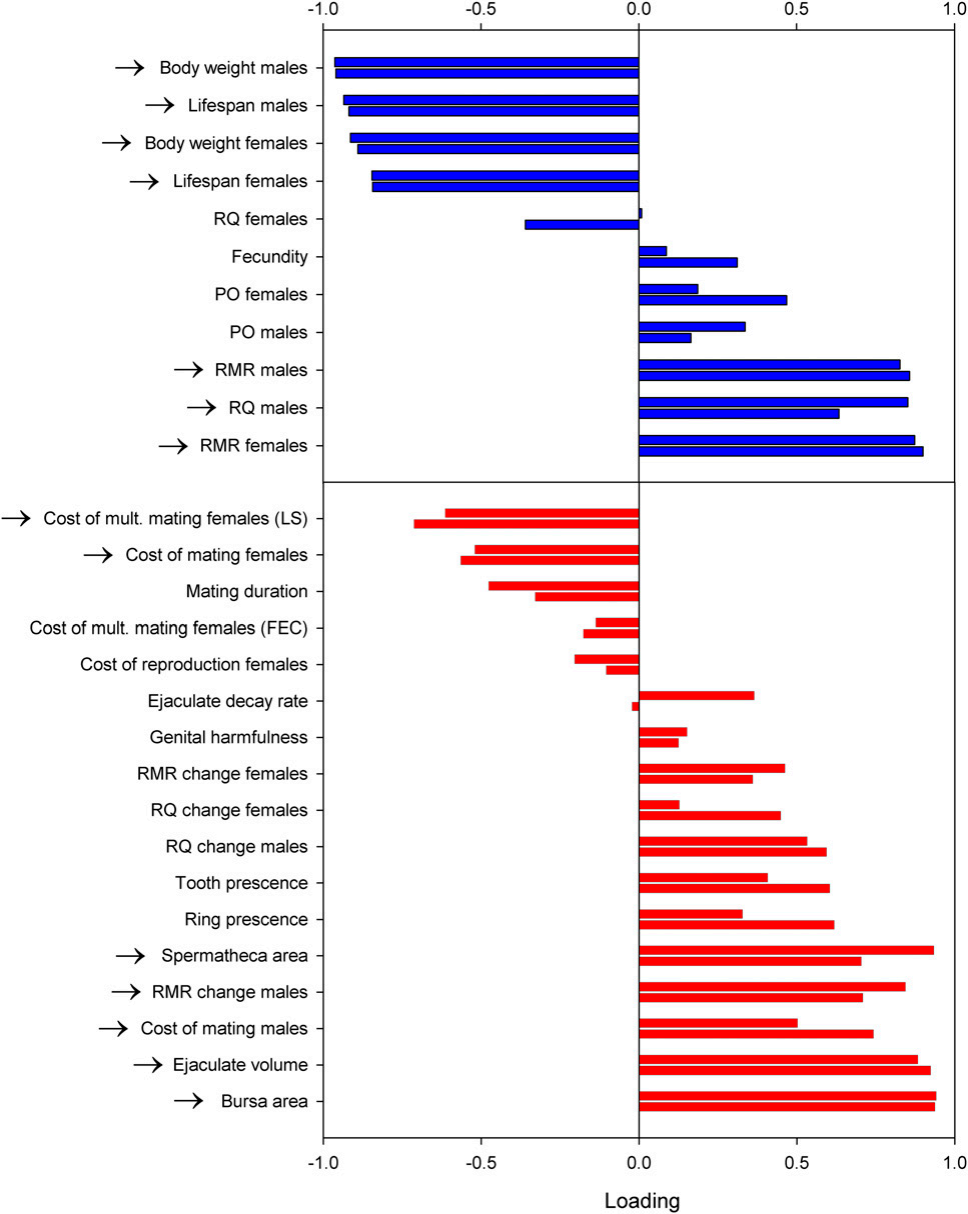
Loadings of all variables on the first and significant principal component of variation in life history (*top*, blue bars) and the economics of mating (*bottom*, red bars) across species. For each variable, the lower bar shows loading based on the analyses of tip data and the upper bar based on the analyses of PICs. Arrows in front of variable names indicate statistically significant (i.e., *P* < 0.05) loadings.

The life history variables showed a single significant PC1, whether assessed by species-level data (*P*_perm_ < 0.001; λ = 5.69; 51.7%[95% CI: 43.3% to 66.1%] of total variance) or by PICs (*P*_perm_ < 0.001; λ = 5.68; 51.8% [95% CI: 45.2% to 67.6%] of total variance). Inspection of the significant loadings on PC1 (Fig. 2) revealed a major axis of correlated life history evolution clearly consistent with evolution along a POLS largely shared by the sexes, characterized by low/high metabolic rate, long/short life span, and large/small body size. A higher RQ (a metabolism less dominated by pure fat oxidation) was also significantly associated with a faster lifestyle, but only so in males. The pattern of covariation among variables was very similar indeed in the species level and in the PICs matrices (Fig. 2), as might be expected given the relatively weak phylogenetic signal.

The reproductive variables also showed a significant PC1 for both species-level data (*P*_perm_ = 0.001; λ = 5.70; 33.5% [95% CI: 29.7 to 54.5] of total variance) and for PICs (*P*_perm_ = 0.009; λ = 5.36; 31.5% [95% CI: 30.5 to 53.3] of total variance), and the pattern of covariation was again very similar in the two matrices. Inspection of the significant loadings (Fig. 2) on PC1 showed that the evolution of low costs of mating and multiple mating in females were associated with 1) a high cost of mating in males, 2) a large ejaculate volume, 3) an increase in male metabolic rate after mating, and 4) large female internal reproductive organs. The antagonistic evolutionary relationship between the cost of mating in males and females is particularly noteworthy. For species-level data, the second principal component was also significant (*P*_perm_ = 0.040; λ = 3.60; 21.2% [95% CI: 16.6 to 30.1] of total variance). Here, ejaculate decay rate (−0.77), RMR change in females after mating (−0.78), and mating duration (0.71) showed significant loadings on PC2, such that species with short matings also showed a high metabolic response to mating in females and a rapid internal processing of the ejaculate, suggesting that metabolic activity is required for ejaculate processing in females.

### Correlated Evolution between Life Histories and the Economics of Mating.

One of our main goals was to characterize and test for concerted evolution between life history traits on one hand and reproductive traits and the economics of mating on the other. However, our multivariate data show a high variables-to-data ratio (i.e., the *P* > n problem) as well as signs of multicollinearity. We thus assessed the overall multivariate pattern of covariation between the two sets of variables by partial least squares correlation (PLSC) analysis. This analysis aims to characterize the multivariate relationships between two matrices by deriving pairs of latent variables (covariance dimensions) where the two matrices play a symmetrical and similar role, which maximizes the covariance between the latent variables. PLSC is appropriate for handling datasets showing the issues at hand in our case (88). All data were centered and standardized prior to analysis. We performed PLSC analyses using TExPosition v.2.6.10.1 (89) and data4PCCAR v.0.1.0 (90) using default settings in R v.4.1.2 (87). We chose this analytical route as it allowed us to explicitly test models and characterize latent variables using resampling statistics (permutation test of eigenvalues and bootstrapping of loadings, here using 10,000 iterations). The analyses were performed with both species-level data and with PICs to control for shared ancestry.

**Fig. 3.**
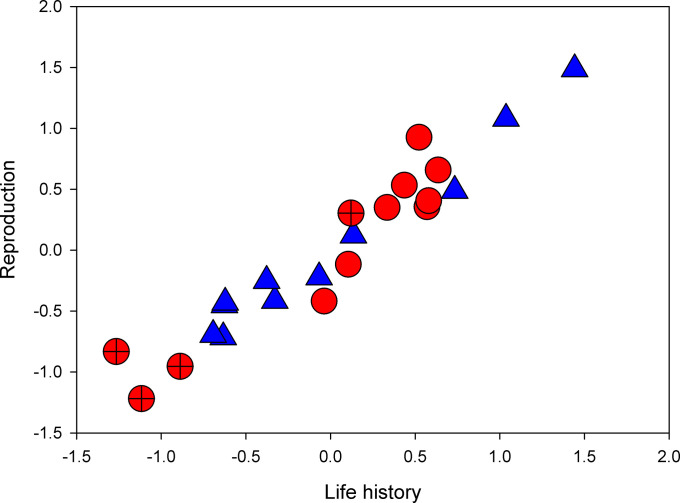
Ordination of the 12 species along the first pair of latent variables describing significant covariation between life history (11 traits) and the economics of mating (17 traits) from PLSC analyses. Red circles denote species values (crosses represent species that utilize trees as hosts), and blue triangles denote PICs. See Fig. 4 for loadings on these vectors.

**Fig. 4.**
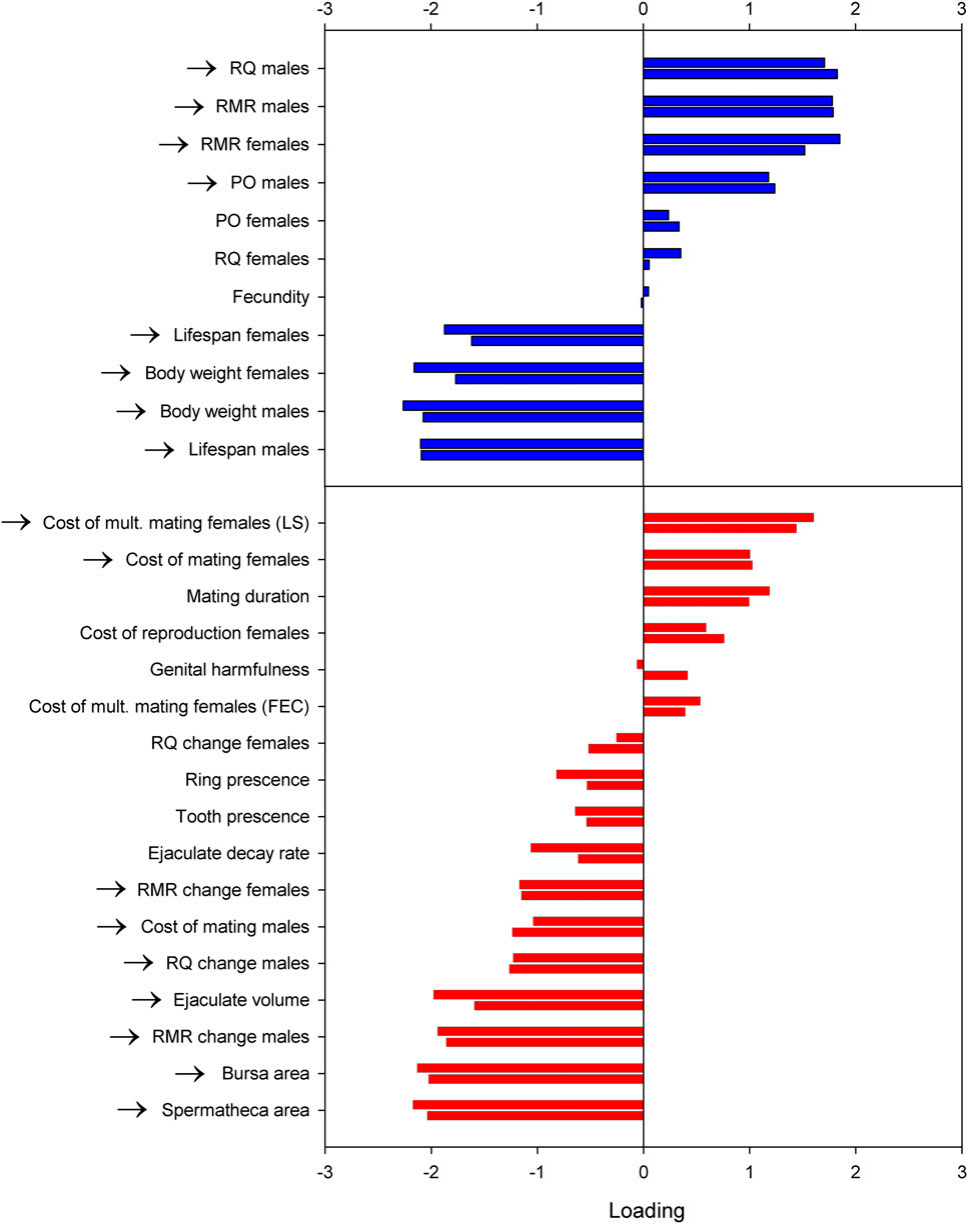
Loadings of all variables on the first and significant pair of latent variables from PLSC analyses describing covariation between life history (*top*, blue bars) and the economics of mating (*bottom*, red bars) illustrated in Fig. 3. For each variable, the lower bar shows loading based on the analyses of tip data and the upper bar based on the analyses of PICs. Arrows in front of variable names indicate statistically significant (i.e., *P* < 0.05) loadings.

For the species-level data, the PLSC yielded a first significant (*P*_perm_ = 0.001) covariance dimension, which accounted for 77% of the covariance between the two matrices (Fig. 3). None of the higher order dimensions were significant (all *P*_perm_ > 0.184). A mirror analysis using PICs showed very similar results. Again, the PLSC yielded a first significant (*P*_perm_ < 0.001) covariance dimension, which accounted for 78% of the covariance between the two matrices (Fig. 3), while none of the higher order dimensions were significant (all *P*_perm_ > 0.312). Hence, the seed beetles studied here showed significant correlated evolution between life history traits and the economics of mating. Inspection of the significant loadings along the first covariance dimension showed a very similar pattern for phylogenetically uncontrolled and phylogenetically controlled data (Fig. 4). Briefly, the main pattern of correlated evolution that emerged from these analyses is one where relatively fast POLS, characterized by small size, short life span, high metabolic rate, and a high male RQ, was associated with a small ejaculate size, a high cost of mating for females, a low cost of mating for males, relatively small female reproductive organs, and a lowered change in metabolic rate as a result of mating in both sexes.

### Specific Models of Trait Evolution.

The dimensionality and richness of our dataset precludes tests of correlated evolution between all possible trait combinations. Yet, our main predictions as well as previous experimental research within species in this group of insects have identified a few key traits that make it possible to erect six specific, relevant, and testable models of trait evolution using an a priori hypothesis-driven phylogenetic generalized least squares (PGLS) model. These models (*SI Appendix*, Table S4) should be seen as a complement to the overall multivariate patterns described above.

**Fig. 5.**
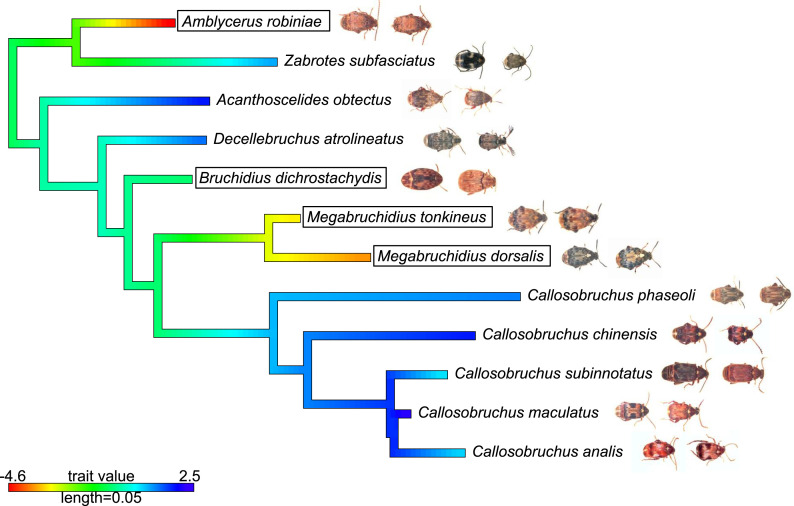
Tip states and ancestral state reconstruction of pace of life (PC1 of life history variables; Fig. 2) for the seed beetle species included. Values at nodes and branches were reconstructed using a maximum-likelihood ancestral character reconstruction based on a Brownian motion model of evolution. Species with bordered names utilize legume trees as hosts; the others utilize herbs as hosts. Pictures illustrate females (left) and males (right) of all species (not to scale).

**Fig. 6.**
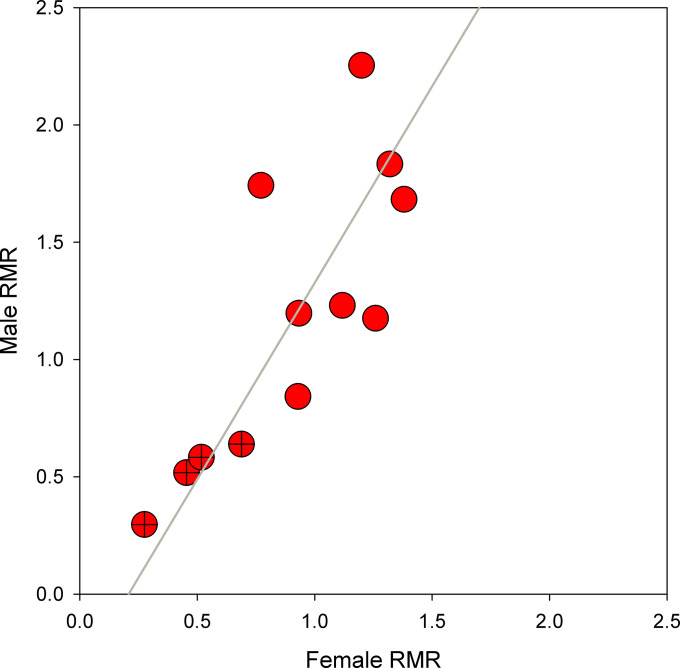
Mass-specific male and female metabolic rates for all 12 species. Crossed circles denote species that utilize trees as hosts, and line represents the slope of a reduced major axis regression, illustrating the more rapid evolution of metabolic rate observed in males.

*1.* ***The economics of mating in females*.** Large male ejaculates are known to affect females positively in this group (80, 91), while injurious male genitalia have been shown to affect females negatively (66–69). Females show a series of adaptations related to mating, including investment in phenoloxidase (PO) to allow healing of internal wounds caused by male genitalia (92) and an enlarged internal reproductive tract (67, 93). In addition, the oval rings in the wall of the bursa copulatrix of some species (94), enclosing an area strongly enriched with trachea, have been suggested to be involved in ejaculate processing (95, 96). This is supported by the fact that species with such rings here showed a larger ejaculate size than those without (*t*_8.15_ = 3.50, *P* = 0.008). A model of the evolution of cost of mating in females using these five predictors showed significant independent negative effects of both ejaculate size and the presence of rings, but not of the other three variables. The addition of female body size to this model did not improve model fit (LLR_1_ = 0.39, *P* = 0.533). An analogous model, instead using the cost of multiple mating as response, showed a significant positive effect of the harmfulness of male genitalia and a marginally nonsignificant negative effect of bursa size. Again, the addition of female body size to this model did not improve model fit (LLR_1_ = 3.62, *P* = 0.057) and did not alter the effects of any other variable.
  These models thus provide support for previous findings in this group of insects: They confirm that the size of the male ejaculate, the harmfulness of male genitalia, the presence of rings in the wall of the bursa, and, possibly the size of the bursa all show correlated evolution with the economics of mating in females in the manner predicted. However, these different predictors apparently affect the economics of mating in females to different extents depending on whether females mate only once or multiply.
*2.* ***The economics of mating in males***. Males of many seed beetles produce and transfer a very large ejaculate at mating, and mating carries substantial costs to males (60, 64). In one of the species studied here, these costs seem to derive primarily from the metabolic costs of ejaculate production, whereby RMR was elevated and RQ depressed as a result of mating (65). It has also been suggested that PO may be involved in alleviating the costs of mating in males, even if male PO levels are very low (92). A model of the evolution of cost of mating in males using these four predictors confirmed that the cost of mating in males indeed increases significantly with ejaculate size; however, it did not detect significant independent effects of PO in males or of RMR or RQ change after mating in males, although the effects were in the predicted direction. The addition of male body size to this model did not improve model fit (LLR_1_ = 0.91, *P* = 0.339).
*3.* ***Correlated evolution between the economics of mating in males and females***. The multivariate analyses provided evidence for a negative association between the costs of mating in the sexes (Fig. 2). To assess whether this was mediated by independent effects of ejaculate volume and genital harmfulness, we predicted the cost of mating in females by cost of mating in males, ejaculate size, and genital harmfulness. For cost of mating in females, this showed independent effects only for ejaculate volume (*t* = 4.26, *P* = 0.003). We conclude that the negative correlated evolution seen between the costs of mating in males and females is primarily caused by the evolution of ejaculate size.
*4.* ***Female investment in PO activity***. The PO system is a major defense system in invertebrates that plays a critical role in a variety of immune reactions and is involved in basic life history trade-offs (97–99). However, PO activity is much higher in females than in males in seed beetles (93), and Bagchi et al. (92) showed that, remarkably, female investment in PO in seed beetles to a large extent represents a counteradaptation to cope with internal injuries caused by harmful male genitalia. We were thus interested in assessing the extent to which female PO activity reflects concerted evolution with life histories versus mating interactions. We assessed this in a model of female PO activity using the following four predictors: male genital harmfulness (92), female life span, female lifetime fecundity, and female RMR. Here, the addition of female body size significantly improved model fit (LLR_1_ = 4.69, *P* = 0.030), so it was also included in the inferential model. Our model, however, showed significant effects only of male genital harmfulness. This implies that, remarkably, the evolution of female PO activity is more closely associated with immune challenges associated with mating than with these basic aspects of their life history.
*5.* ***The role of ecology in life history evolution***. We set out to test the key hypothesis that host plant type drives the evolution of life history syndromes in this group of insects. This was tested in PGLS models using the dichotomous variable tree/herbaceous host to predict the two latent variables summarizing life histories discussed above. These models showed a tight correlated evolution between host type and both PC1 of life history (β = −4.31 [95% CI: −5.68 to −2.94], *t* = 6.17, *P* < 0.001) (Fig. 5) and PLSC1 of life history (β = −1.23 [95% CI: −1.63 to −0.84], *t* = 6.13, *P* < 0.001). Thus, as species evolve to utilize trees as hosts, they also evolve a considerably slower POLS with reduced metabolic rate, longer life span, and larger body size. Additional models showed that host type also predicted evolution of the economics of mating, although to a somewhat lesser extent (PC1: *t* = 2.23, *P* = 0.049; PLSC1: *t* = 2.95, *P* = 0.015).
*6.* ***Sex-specific evolution of metabolic rate***. To test whether male metabolic rate evolves more rapidly than female metabolic rate, we inspected the concerted evolution of sex-specific RMR to test whether male RMR shows >1:1 scaling with female RMR. We regressed male RMR on female RMR using PICs by both i) standard least squares (LS) regressions forced through the origin and ii) reduced major axis regression. The first approach showed a significantly >1:1 scaling (β = 1.73, 95% CI_boot_: 1.18 to 2.69) as did the second (β = 2.17, 95% CI_boot_: 1.32 to 3.49) (Fig. 6). Hence, male RMR evolves more rapidly than female RMR, consistent with the observation of a high and significant phylogenetic signal of RMR in females but not in males (*SI Appendix*, Table S3). We note that neither body size (β = 1.02, 95% CI_boot_: 0.85 to 1.47) nor life span (β = 0.70, 95% CI_boot_: 0.49 to 1.32) showed such nonunity scaling (using the first approach above). To compare the scaling relationship between RMR on one hand and life history and reproduction on the other in the sexes, we regressed sex-specific RMR on the four latent variables discussed above using reduced major axis regression and compared the slopes in males and females using likelihood ratio tests. This showed that the slope in males was significantly steeper than in females for both PC1 (χ^2^_1_ = 4.98, *P* = 0.026) and PLSC1 (χ^2^_1_ = 4.06, *P* = 0.044) for life history variables. The slope was steeper in males than in females also for the economics of mating, but not significantly so (PC1: χ^2^_1_ = 2.03, *P* = 0.154; PLSC1: χ^2^_1_ = 2.93, *P* = 0.114). These analyses thus show that although RMR is closely integrated with POLS in both sexes, male RMR shows a more rapid correlated evolution with POLS.

## Discussion

Metabolic rate has recently gained a central position in life history evolution (16, 17), but unraveling its role as a nexus has proven challenging (35). Our unique sex-specific experimental common garden data, analyzed across multiple species that varied extensively in all traits measured, were not only consistent with a central role for RMR in life history evolution but also showed that RMR is predictably integrated with the effects of ecology on male–female coevolution. Here, we highlight a series of specific insights. First, RMR indeed showed a tight correlated evolution with life history traits describing variation along a POLS. Second, evolutionary transition to ecological conditions less characterized by resource limitation was associated with the evolution of increased RMR and a more rapid POLS. Third, and perhaps most importantly, the economics of mating showed correlated evolution with RMR, POLS, and ecology in a manner consistent with predictions based on our understanding of sex-specific selection in this group of insects. Fourth, our analyses reveal a more rapid evolution of male RMR than female RMR across species, in line with the common assumption that selection on RMR is stronger in males than in females. Below, we discuss each of these points in more detail.

Comparative studies typically face a trade-off between the number of taxa included and the quality of the data. In particular, data gathered in different environments or using different methods can blur results and introduce bias (35, 38). We employed a strategy in which standardized data were gathered under common garden conditions for a relatively restricted number of species, and our analyses successfully revealed a close integration of RMR and key life history traits. This is consistent with a central place for physiological processes in life history evolution, where the rate of resource processing dictates POLS (16, 17). The fact that evolution under ecological conditions with higher resource limitation (i.e., the usage of perennial trees as hosts) was associated with a lower RMR and slower POLS provides a mechanistic basis for the pattern of correlated evolution and thus adds substantial support for a central and causative role of metabolic constraints. We note that previous experimental evolution studies have provided evidence for a central role of RMR in POLS evolution in one of the species studied here. This is true for studies of physiological (100), phenotypic (83), as well as genetic (101) differentiation under divergent life history evolution. Although our efforts were substantial and sufficient to detect the integration between RMR and POLS, additional traits are no doubt part of POLS in this group of insects but were not specifically assayed here. This would, for example, include behavioral traits such as general activity (19), a possibility supported by a positive correlation between a crude measure of mean activity (movement during our metabolic rate assays) and RMR across species in both females (*r* = 0.62) and males (*r* = 0.80). Similarly, additional life history traits such as juvenile development time and age at reproductive maturity are likely integrated as well, again tentatively supported by the fact that species showing a slow POLS (e.g., *Megabruchidius* spp.) have both longer juvenile development times and reproduction at later adult ages than species showing a faster POLS (e.g., *Callosobruchus* spp.) (60).

Our analyses imply that ecology ultimately constrains resource use and metabolic rate, which sets the stage not only for life history evolution but also for the evolution of reproductive interactions and mating systems. A dramatic example of the latter are honey locust beetles (*Megabruchidius* spp.) studied here, which utilize legume trees in the genera *Gleditsia* and *Styphnolobium* as hosts (102), where infestation rates of seeds are very high (52). Honey locust beetles have evolved a slow POLS and show sex-role reversal: Females search for and actively court males who are choosy, and females can live for several months and continue to produce eggs only if mated to males with regular intervals (59, 60). Male ejaculates are large and nutritious, and each ejaculate allows the female to produce about 10 additional eggs (76). Intense resource and reproductive competition (80) has thus resulted in females that essentially forage for matings [*sensu* (103)] in this genus.

**Fig. 7.**
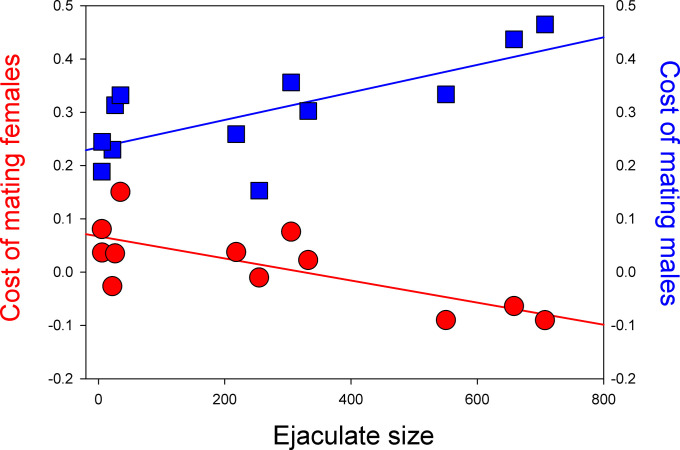
The relationship between ejaculate size and sex-specific cost of mating across all 12 species (red circles: females, blue squares: males). The cost of mating shows negative correlated evolution with ejaculate size in females but positive in males.

In many insects, males transfer substances in the ejaculate that provide benefits for females (104). The evolution of such nuptial gifts has been suggested to be closely linked with ecology, life history evolution, and the economics of mating, but our understanding of this integration is incomplete (44). We show that the cost of mating to males and females exhibits negative correlated evolution: As the cost of mating in males increases, the cost of mating in females decreases (Fig. 7). Although a negative relationship between male and female investment in reproduction is a long-held tenet in sexual conflict theory (105) with scattered support in the literature (9), we know of no previous direct demonstration of this sexually antagonistic relationship. This is significant primarily because it shows that the evolution of the economics of mating is constrained, such that adaptations that decrease the cost of mating in one sex may simultaneously increase the cost of mating in the other. As such, this finding provides evidence for the sometimes conflicting reproductive interests of the sexes (9). Moreover, we show that male ejaculate size forms the main common denominator for the economics of mating in both sexes, and this is linked to ecology, RMR, and POLS. It is worth noting that the evolution of large ejaculates was associated with an increase in metabolic rate after mating in both sexes, presumably because of costs of ejaculate renewal in males (65) and the metabolic processing of ejaculate resources in females. In slow POLS species adapted to conditions of sustained pre- and postmating reproductive competition, males have evolved to allocate resources to enlarged ejaculates with consequential effects on the economics of mating and the mating system. Experimental assays have shown that sexual selection is indeed stronger in such species in both sexes (54), and an experimental evolution study confirmed that intensified reproductive competition among males leads to the evolution of larger ejaculates, which benefits females (80). In fast POLS species where sexual selection is stronger in males than in females (54), males have adapted to “live fast, die young” (83, 106) and instead apparently allocate relatively more resources to competing demands such as general activity and mate search (81). Finally, we note that the negative correlated evolution between the cost of mating in males and females ultimately derives from the fact that females benefit from larger ejaculates, and this pattern of coevolution of the economics of mating may not be manifest in groups where this is not true.

A distinct but noteworthy implication of the intimate evolutionary links between ecology, life history, mating system, and sexual selection relates to our ability to disentangle the independent effects of ecology (107) and sexual selection (108) on the rate of speciation in phylogenetic comparative studies. Such studies have yielded mixed results (109, 110). If the strength of resource and reproductive competition covaries and both have positive effects on net speciation rates, the role of one process will confound the role of the other. This will complicate empirical efforts but also blur the distinction between modes of speciation (111).

Although the integration of metabolism and life history traits was largely shared between the sexes, two notable aspects were sex specific. First, a higher RQ was associated with a faster POLS in males, but not in females. Seed beetles are to a large extent capital breeders, and their relatively low adult RQ (ca. 0.7) reflects the fact that they primarily metabolize fat deposits acquired during their juvenile phase. Our results suggest that the sexes achieve a higher RMR in partly distinct ways: While POLS was not associated with respiratory substrate use in females, male RQ was more variable and was integrated with POLS, such that a high RMR in males was associated with a metabolism less dominated by pure fat oxidation. Males in fast POLS species thus use proteins and/or carbohydrates as additional metabolic substrates to a larger extent, likely reflecting selection for a live fast, die young strategy. This is supported by the fact that seed beetle populations experimentally evolved for a fast POLS show an elevated RQ (83). We note that evolved differences in metabolic substrate use between the sexes may translate into sex-specific optima in diet composition (112, 113).

Second, males are often predicted to have a higher optimal RMR than females in polyandrous taxa (27, 28), and sexual dimorphism in estimates of RMR offers some support for this general prediction (30, 114). This implies that the manifestations of ecology on RMR and POLS may differ in the sexes, and we predicted and observed that increased resource availability was associated with a more rapid evolution of RMR in males than in females. This was reflected in a more rapid correlated evolution between RMR and life history in males than in females. Such differences between the sexes in the evolution of metabolic rate offer an additional and general mechanism for the evolution of sexual dimorphism in life histories (26) and male–female coevolution. Incidentally, this scenario would generate links between ecology and sexual dimorphism, which are distinct from those generated by ecological character displacement between the sexes (115). The generality of our finding is difficult to assess at this point, as very few studies have measured sex-specific RMR in multiple species under common garden conditions. Those that have, however, are supportive. Berrigan and Hoang (116) estimated RMR in nine species of *Drosophila* flies, Krasnov et al. (117) did the same in seven species of fleas and Cullum (118) in six species of lizards. Reduced major axis regression of their data of male metabolic rate on female metabolic rate across species showed estimated slopes >1 in all cases (β = 1.45, 95% CI_boot_: 0.95 to 2.84; β = 1.61, 95% CI_boot_: 0.71 to 5.15; and β = 1.10, 95% CI_boot_: 0.91 to 2.15, respectively), in line with a more rapid evolution of RMR in males than in females.

Although our data are based on extensive experimental efforts and although several key evolutionary paths have previously been validated by experimental evolution, our study is ultimately a comparative one and to some extent suffers from inevitable inferential limitations in terms of causation (37, 38). To strengthen the casual inferences made here, well-replicated experimental evolution studies would be very valuable. Such long-term experiments would need to quantify the cascading effects of varying the strength of resource competition on sex-specific life histories, metabolic phenotypes, the economics of mating, and mating system parameters. Further, in systems where this is possible, quantitative analyses explicitly based on mass–energy balance considerations and the bio-physical constraints that dictate resource acquisition and allocation (119–121) could provide further insights.

## Conclusions

We document substantial evolution of metabolic rate in a well-delineated and homogenous group of insects: mass specific RMR varied across species by a factor of five in females and eight in males. The evolution of RMR was embedded within a complex of life history traits, well described by a POLS. Moreover, RMR and life history showed correlated evolution with ecology (host use). This trajectory was concerted with the evolution of sex-specific reproductive traits in both sexes, and the economics of mating thus showed correlated evolution with life history evolution. The cost of mating showed sexually antagonistic coevolution, with the economic outcome of mating being contingent upon ecology. Overall, our results support the tenet that resource competition dictated by ecological factors has direct effects on metabolic processes, which, in turn, has predictable cascading effects on life history evolution, the economics of mating, and male–female coevolution.

## Materials and Methods

### Species and Rearing.

We performed parallel experimental studies of 12 seed beetle species. Adults of these beetles are capital breeders and do not require food or water to reproduce successfully, and aphagous conditions were used in all experiments. Both sexes mate multiply and reproduce soon, if not immediately, after adult emergence from host beans. Females then lay eggs on or near their host legume beans, and the larvae complete their life cycle and pupate inside the bean. Although the species studied here thus share a common basic ecology, they differ in host plant use (48, 122) and mating system (54). The species included (*SI Appendix*, Fig. S5) were *Callosobruchus maculatus* (A), *Callosobruchus analis* (B), *Callosobruchus subinnotatus* (A), *C. phaseoli* (B), *C. chinensis* (C), *Acanthoscelides obtectus* (D), *Decellebruchus atrolineatus* (A), *B. dichrostachydis* (E), *Zabrotes subfasciatus* (D), *A. robiniae* (F), *Megabruchidius tonkineus* (F), and *M. dorsalis* (F), where letters denote the preferred native host also used here for rearing (A: *Vigna unguiculata*, B: *Vigna radiata*, C: *Vigna angularis*, D: *Phaseolus vulgaris*, E: *Dichrostachys cinerea*, and F: *Gleditsia triacanthos*) (122). The hosts A–D are annual herbs, and E–F are perennial trees. All beetles were reared in 1-L stock jars in climate cabinets in the laboratory at Uppsala University, at 25 to 29 °C, 55 to 70% relative humidity (RH), and a 12L:12D light regimen. Jars were provided with ample host beans, and the census adult population sizes were approximately *n* = 300 to 400. We conducted four major experiments and extracted species-specific data on 11 life history traits and 17 traits related to the economics of mating in all 12 species. A detailed description of these experiments is available in *SI Appendix*, *Supplementary Materials and Methods*, and a list of all traits is given in *SI Appendix*, Fig. S1.

We used microrespirometry to derive measures of metabolic parameters in virgins and mated individuals of both sexes. These were used to calculate RMR and RQ. We used RMR and RQ in virgin individuals as the baseline and the difference in RMR and RQ between virgins and mated to quantify mating-induced changes in metabolism.

To quantify the cost of mating in males, males were randomly assigned to one of two treatments: one in which males were precluded from reproductive interactions and one in which males mated and competed against other males freely. The cost of mating to males was then estimated as the proportional reduction in life span induced by mating.

Our assessment of the cost of mating and reproduction in females exploited the fact that females do not lay eggs in the absence of host beans. This allowed the experimental separation of the economic effects of mating from those of reproduction. Females were thus assigned to (A) no mating or reproduction, (B) mating once but no reproduction, (C) mating once with reproduction, or (D) multiple mating with reproduction. Egg production (fecundity) and life span were determined for all females. We then used these data to estimate the proportional change in life span induced by mating (the cost of mating), the reduction in life span resulting from egg production (cost of reproduction), and the cost of multiple mating in females (manifested both in terms of changes in life span and lifetime fecundity). For cost metrics, a positive value represented a cost, and a negative represented a benefit.

To determine postmating ejaculate processing inside females, females were flash frozen at either 0, 4, 8, 24, 30, or 48 h after mating. Here, mating duration was recorded, and females were subsequently dissected under a microscope. We used these data not only to estimate male ejaculate volume and the rate at which the ejaculate was processed by females but also to characterize several internal reproductive anatomical traits (*SI Appendix*, Fig. S1).

To compensate for potential effects of phylogenetic independence, we tested for correlated evolution both through the use of PICs and through PGLS models of trait evolution.

## Supplementary Material

Supplementary File

Supplementary File

Supplementary File

Supplementary File

## Data Availability

Data reported in this paper is included in the *SI Appendix*. All experimental data have been published in Mendeley Data and are publicly available (123).
