# Supplementary material for: Concerted evolution of metabolic rate, economics of mating, ecology, and pace of life across seed beetles
Source: Proc Natl Acad Sci U S A. 2022 Aug 9;119(33):e2205564119. doi: 10.1073/pnas.2205564119 (PMC9388118; doi:10.1073/pnas.2205564119)
Supplement: Supplementary File [file pnas.2205564119.sapp.pdf]

PNAS
www.pnas.org

Supplementary Information for

Concerted evolution of metabolic rate, economics of mating, ecology and pace-of-life across seed beetles

Göran Arnqvist, Johanna Rönn, Christopher Watson, Julieta Goenaga, Elina Immonen

Corresponding author: Göran Arnqvist
Email: Goran.Arnqvist@ebc.uu.se.

This PDF file includes:

SI Materials and Methods
SI References
Figures S1 to S5
Tables S1 to S4
Datasets S1 to S4

Supplementary Information Text

Supplementary Materials and Methods

Metabolic rate. To derive measures on metabolic parameters in the two sexes across all species, and to estimate how this differs between mated and virgin individuals, we measured resting metabolic rate (RMR) and respiratory quotient (RQ) of single beetles using a Sable Systems (Las Vegas, NV, USA) micro-respirometry system (1). This system pumps air at a very precisely regulated flow rate through a sealed chamber containing the animal. Downstream gas analyzers are then used to measure the amount of CO_2 produced and O_2 consumed by the beetles, and these measures then provide estimates of metabolic parameters. Briefly, a LiCor 7000 infrared gas analyzer (Lincoln, NE), a Sable systems FC-2 differential oxygen analyzer (Las Vegas, NV), and a RH-300 water vapor pressure meter (Sable Systems) were attached to two Sable Systems RM8 eight-channel multiplexers. Respirometry chambers (RC-M, Sable Systems; ø = 2 cm, length = 4 cm) were housed inside a Sanyo MIR-153 incubator with temperature held at 30°C. One of the 16 chambers was left empty and was measured at repeated occasions during the recordings to control for instrumental drift. Inflowing air was pumped using a SS-4 pump (Sable Systems) and flow was regulated to 50 mL/min using a Model 840 mass flow control valve (Sierra Instruments, Monterey, CA). To allow for a compensation of varying degrees of activity, each respirometry chamber was placed in an activity detector (AD-2; Sable Systems) connected to a data acquisition interface (Quick-DAQ; National Instruments, Coleman Technologies, Newton Square, PA, USA). This uses reflective infrared light technology to provide a precise and continuous measure of locomotor activity of the subjects in each chamber during the entire session. All analogue input data were acquired at 1 Hz via a UI2 analog-digital interface (Sable Systems). Data acquisition and data analyses were performed in ExpeData Pro 1.5.6 (Sable Systems).

The respirometry system was set up in stop-flow mode (1), in which each chamber was sealed for a period of 60 min and then flushed for a period of 150 sec. Each cycle (through all chambers) lasted for 62 min and 30 sec, and each measuring session lasted approx. 15 hrs (5 PM to 8 AM). This resulted in 5 cycles/readings for each individual, of which the first was

discarded as a burn-in. Thus, each observation consisted of four repeated measures of the amount of CO_2 produced and O_2 consumed during 60 min by an individual beetle and the cumulated amount of activity performed during this time.

All individuals were ca 24 hrs old virgins at the start of the experiment. Half of all individuals were allowed to mate immediately before being placed in a respirometry chamber while the other half remained virgin. All individuals were weighed to the nearest 0.01 mg using an electronic microbalance (Sartorius Genius ME 235P-OCE) prior to the experiment, and all males in the mated treatment were weighed both before and after mating to determine ejaculate weight (as the difference in body weight). Our experimental design thus had 48 cells (12 species × 2 sexes × 2 mating status) and the average sample size per cell was 15.7 individuals (total N = 753).

Measures of metabolic rate are often confounded by an unknown degree of variation in activity during recording (1). Here, we used activity-compensated measures of CO_2 and O_2 for all downstream analyses. For each cycle, species and sex, we regressed both CO_2 and O_2 on activity and added the residual to the respective intercept. This yields the value expected under zero activity for all observations (i.e., resting metabolic rate), and will equal the observed value for all individuals showing zero activity. The overall correlation between uncompensated and compensated CO_2 and O_2 values was r = 0.93, suggesting that on average 14% of total variance was due to variation in activity across individuals. We assessed sex and species specific scaling in metabolic rate by fitting the function $CO_2 = c + \text{body mass}^m$, by means of non-linear regression using data on virgin individuals. The average exponential scaling coefficient was $m = 1.12$ in females and $m = 1.17$ in males, but in no species did the exponential scaling coefficient differ between the sexes within species ($P > 0.054$ in all cases). In only four out of 24 cases did the 95% CI of m not overlap with $m = 1$ within species. Here, we thus follow convention (1) and use activity compensated values of CO_2 and O_2 per mg body weight (i.e., mass-specific RMR) as our measure of RMR and the ratio CO_2 produced /O_2 consumed as our measure of RQ in all comparative analyses.

An analysis of the full data set showed a strong general effect of mating status on metabolism, which differed in magnitude between species and between sexes. Here, we

thus used the marginal mean per cell of our design, averaged over all four cycles, to characterize the species, sex and treatment specific metabolic metrics. For each sex, we derived four species specific measures of weight specific metabolic parameters. (1) RMR and (2) RQ in virgin individuals, to provide baseline metabolic parameters. (3) RMR_{mated} – $RMR_{virgins}$ and (4) RQ_{mated} – $RQ_{virgins}$ were used to characterize mating-induced changes in metabolism.

Cost of mating in males. Virgin males and females were collected upon emergence from beans and isolated individually. Males were randomly assigned to one of two treatments, one in which males were precluded from reproductive interactions and one in which males mated and competed against other males freely. In treatment A, each male was placed alone in a petri dish for life. In treatment B, three males and three females were introduced together for life in a petri dish. In both treatments, males were weighed prior to the experiment and all dishes were provided with an ample supply of beans. We performed spot checks at least once per 24 hrs to determine male life span. We conducted 16 - 20 replicates per treatment level and species (total N = 457 males).

The data from this experiment was used to estimate two metrics. (1) Virgin male life span is represented by mean life span in group A. (2) The cost of mating to males was estimated as the proportional change in life span induced by mating interactions, i.e. $(\bar{A} - \bar{B})/\bar{A}$ where letters denote mean life span in the two groups.

Cost of mating and reproduction in females. Females of some species of seed beetles suffer costs of male harassment (2,3) so in order to isolate the economic effects of mating in females we did not employ male-female cohabitation but staged all matings. Virgin males and females were isolated individually upon emergence and all individuals used were young and had just entered adult reproductive maturation when assigned to a treatment (i.e., 1-3 days old in all species but in *M. dorsalis, M. tonkineus* and *A. robiniae* where individuals were 5-7 days old). Females generally do not lay eggs in the absence of host beans and to separate the economic effects of mating itself from the cost of reproduction, females were thus assigned to either of the following four treatments; (A) virgin females were isolated in an empty 9 cm Ø petri dish (i.e. no mating or reproduction), (B) females were mated once and

then isolated in an empty petri dish (i.e. mating once but no reproduction), (C) females were mated once and then isolated in a petri dish containing beans (i.e. mating once and reproduction), (D) females were mated twice (day 1 and 3) and were from the first mating isolated in a petri dish containing beans (i.e. mating multiply and reproduction). Matings were staged by introducing a virgin male to the female. All matings were monitored and males removed from vials following copulation. Females in treatment C and D were provided with a superabundant supply of host beans (21 g). Females were weighed to the nearest 0.01 mg using an electronic microbalance (Sartorius Genius ME 235P-OCE) prior to the experiment, and all males weighed both before and after mating to determine ejaculate weight (as the difference in body weight).

We conducted on average N = 17.6 replicates per treatment level and species (range 8 – 27; total N = 843 females). Female life span (spot checks ≥1 per day) and reproductive effort (the number of eggs dumped in petri dish in treatment A and B, number of offspring produced in treatment C and D) were recorded in all replicates. In treatment groups C and D, a few females that laid fewer than 5 eggs in their lifetime were omitted from further analysis, as they had likely not copulated successfully (N = 54 in total; N = 45 of these laid zero or only a single egg) and inclusion of these would bias our downstream analyses. We then estimated species and treatment level specific average life span and average fecundity for level C and D from this experiment.

For our estimates of the cost of mating and the cost of egg production, the treatment levels A and B should represent female life span in the absence of the costs of egg production. However, some females in A laid a few eggs despite them being virgin (i.e., egg dumping) and some females in B laid a few eggs despite the absence of larval resources (i.e., beans). Because female life span is negatively related to egg production (*SI Appendix,* Fig. S2), the raw life span means are less than ideal. For these two levels, we therefore estimated female life span compensated for egg production for females in treatment levels A and B. For each species, we performed a linear regression of life span on the number of eggs laid, and for each female we then summed the residual with the species specific intercept. For females laying no eggs, this equals their observed life span. For those females that laid a few eggs, this yields the predicted life span that each female should have had if she had laid no eggs.

We then estimated species and treatment level (for A and B) specific average life span using these compensated values. We note that they differed only very marginally from the raw uncompensated estimates (difference on average 3.8%).

The data from this experiment was used to estimate six species-specific metrics. (i) Virgin female life span is represented by mean life span in treatment A and (ii) average life time fecundity by mean fecundity in treatment D. (iii) The cost of mating in females was estimated as the proportional change in life span induced by mating, i.e. $(\bar{A} - \bar{B})/\bar{A}$ where letters denote mean life span in treatment groups. (iv) The cost of reproduction was estimated as the proportional change in life span induced by egg production, i.e. $(\bar{B} - \bar{C})/\bar{B}$ where letters denote mean life span in treatments. The cost of multiple mating in females was estimated by the proportional change between treatments C and D as $(\bar{C} - \bar{D})/\bar{C}$, and we estimated this cost for both (v) mean life span and (vi) mean life time fecundity. For metrics 3, 5 and 6 above, a positive value thus represents a cost to females and a negative a benefit.

Postmating ejaculate processing in females and ejaculate size. Virgin males and females were collected upon emergence from beans and isolated individually. Females were randomly assigned to one of seven treatments (A, B, C, D, E, F and G; *N* = 5 females per treatment). In treatment A, females were flash frozen as virgins. In treatment B, the females were mated with a virgin male and flash frozen immediately after copulation had taken place (time 0). These form our reference females where the entire male ejaculate is preserved within the female reproductive system. In the remaining treatments, the females were mated with a virgin male and then flash frozen at staggered time points after copulation to allow for ejaculate processing (C = 4 hrs, D = 8 hrs, E = 24 hrs, F = 30 hrs, G = 48 hrs). Copulation duration, defined as the time from full male genital insertion to male-female separation, was recorded for all matings. All females were subsequently dissected under a Leica M165 C stereo microscope and several photos were taken of internal reproductive anatomical traits using a Lumenera Infinity 2-5C digital microscope camera, under standardized setting and lighting.

Images were analysed using Infinity Analyze 6.1. The bursa copulatrix (BC) is semitransparent and the area of the ejaculate was recorded as seen through the dorsal wall of the intact BC (*SI Appendix,* Fig. S3). The area of the BC itself was recorded following dorsal slitting and spreading of the BC, placed under a microscope slide. We also recorded the area of the spermatheca, the presence of chitinised oval rings and a tooth on the wall of the BC (*SI Appendix,* Fig. S3) and, finally, the length of the elytra to provide a measure of general female body size.

To characterize the ejaculate processing rate of each species (*SI Appendix,* Fig. S4), we first calculated ejaculate volume based on ejaculate area, using a spherical approximation. Ejaculate volume at time 0 correlated very well with measures of ejaculate weight from the experiment on the cost of mating in females across species ($r = 0.95$) and was here used as our measure of ejaculate size. For ejaculate processing rate, we first divided all measures of ejaculate volume by the species specific mean volume in treatment B (i.e., time 0), thus relativizing ejaculate volume. We then fitted an exponential decay model in each species, with an intercept of 1, using robust nonlinear regression, where relative ejaculate volume = $e^{(-b * \text{time})}$. Here, b is proportional to the rate of decay and represents our measure of ejaculate processing rate.

Additional data. Because investment in immune function may be central in life history trade-offs and because reproductive traits show correlated evolution with immunity in seed beetles (4) we included relevant complimentary data for all 12 species on sex-specific immunity and male genital morphology from Bagchi et al. (4). The data were (i) phenoloxidase (PO) activity of virgin females, (ii) PO activity of virgin males and (iii) the degree of injuriousness of male genitalia.

Phylogenetic comparative analyses. To compensate for potential effects of phylogenetic independence, we used a phylogeny representing the pruned supertree of Kergoat et al. (5), resulting from 15 source trees based on mitochondrial and nuclear genetic data as well as morphology. The topology of this phylogenetic hypothesis is very well supported indeed (5,6). To assess phylogenetic signal in our data, we estimated both Pagel's λ (7) and Blomberg's K (8) for all traits. For both metrics, values of zero indicates that traits have

evolved independently of the phylogeny (i.e., close relatives are not more similar than more distant relatives) while unity represents a strong phylogenetic signal consistent with trait evolution according to a Brownian motion model. We tested for correlated evolution both through the use of PICs (9) and through phylogenetic generalized least squares (PGLS) models of trait evolution (10), using the ML estimate of λ. Phylogenetic comparative analyses were performed using ape v.5.5 (11) and phytools v.0.7-90 (12), in R v.4.1.2 (13).

SI References

1. J. R. Lighton, *Measuring metabolic rates: a manual for scientists*. (Oxford University Press, 2018).
2. G. Sakurai, E. Kasuya, The costs of harassment in the adzuki bean beetle. *Anim. Behav.* **75**, 1367-1373 (2008).
3. L. Gay, P.E. Eady, R. Vasudev, D. Hosken, T. Tregenza, Costly sexual harassment in a beetle. *Physiol. Entomol.* **34**, 86-92 (2009).
4. B. Bagchi, Q. Corbel, I. Khan, E. Payne, D. Banerji, J. L. Rönn, I. Martinossi-Allibert, J. Baur, A. Sayadi, E. Immonen, G. Arnqvist, I. Söderhäll, D. Berger, Sexual conflict drives micro-and macroevolution of sexual dimorphism in immunity. BMC Biol. 19, 1-19 (2021).
5. G. J. Kergoat, A. Delobel, B. Le Ru, J. F. Silvain, Seed-beetles in the age of the molecule: recent advances on systematics and host-plant association patterns. *Research on Chrysomelidae* **1**, 59-86 (2008).
6. M. Tuda, J. Rönn, S. Buranapanichpan, N. Wasano, G. Arnqvist, Evolutionary diversification of the bean beetle genus *Callosobruchus* (Coleoptera: Bruchidae): traits associated with stored-product pest status. *Mol. Ecol.* **15**, 3541-3551 (2006).
7. M. Pagel, Inferring the historical patterns of biological evolution. *Nature* **401**, 877–884 (1999).
8. S. P. Blomberg, T. Garland, A. R. Ives, Testing for phylogenetic signal in comparative data: behavioral traits are more labile. *Evolution* **57**, 717–745 (2003).
9. J. Felsenstein, Phylogenies and the comparative method. *Am. Nat*. **125**, 1-15 (1985).
10. E. P. Martins, T. F. Hansen, Phylogenies and the comparative method: a general approach to incorporating phylogenetic information into the analysis of interspecific data. *Am. Nat.* **149**, 646-667 (1997).
11. E. Paradis, K. Schliep, Ape 5.0: an environment for modern phylogenetics and evolutionary analyses in R. *Bioinformatics* **35**, 526-528 (2019).
12. L. J. Revell, Phytools: an R package for phylogenetic comparative biology (and other things). *Meth. Ecol. Evol.* **3**, 217-223 (2012).
13. RDevelopmentCoreTeam, R: a language and environment for statistical computing. http://www.R-project.org/ (2021).

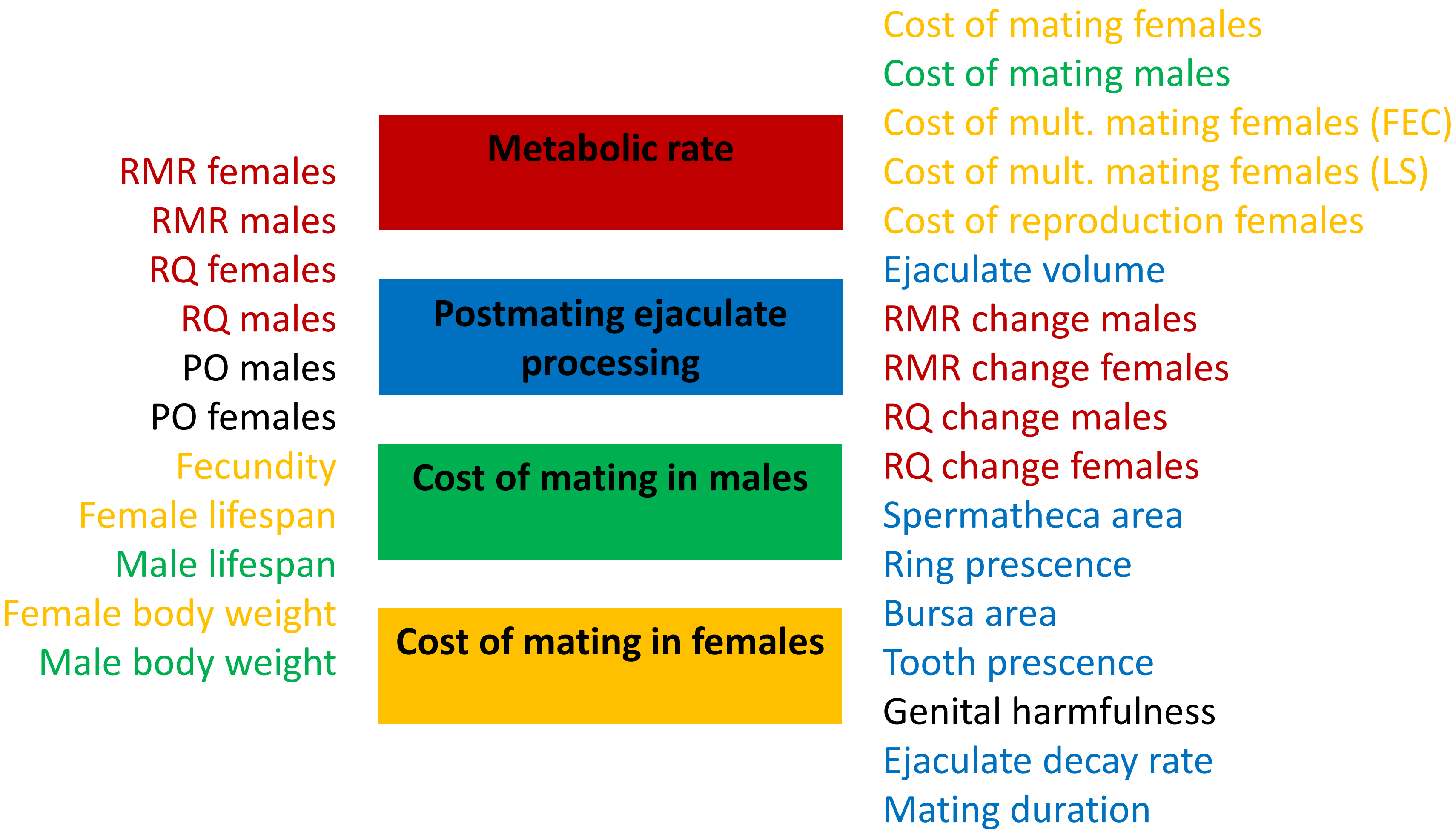

Figure S1. Overview over all 11 life history traits (left column) and 17 reproductive variables (right column), color coded to match the experiment (center) from which they were extracted. Three variables (in black font) were gained from Bagchi et al. (2021).

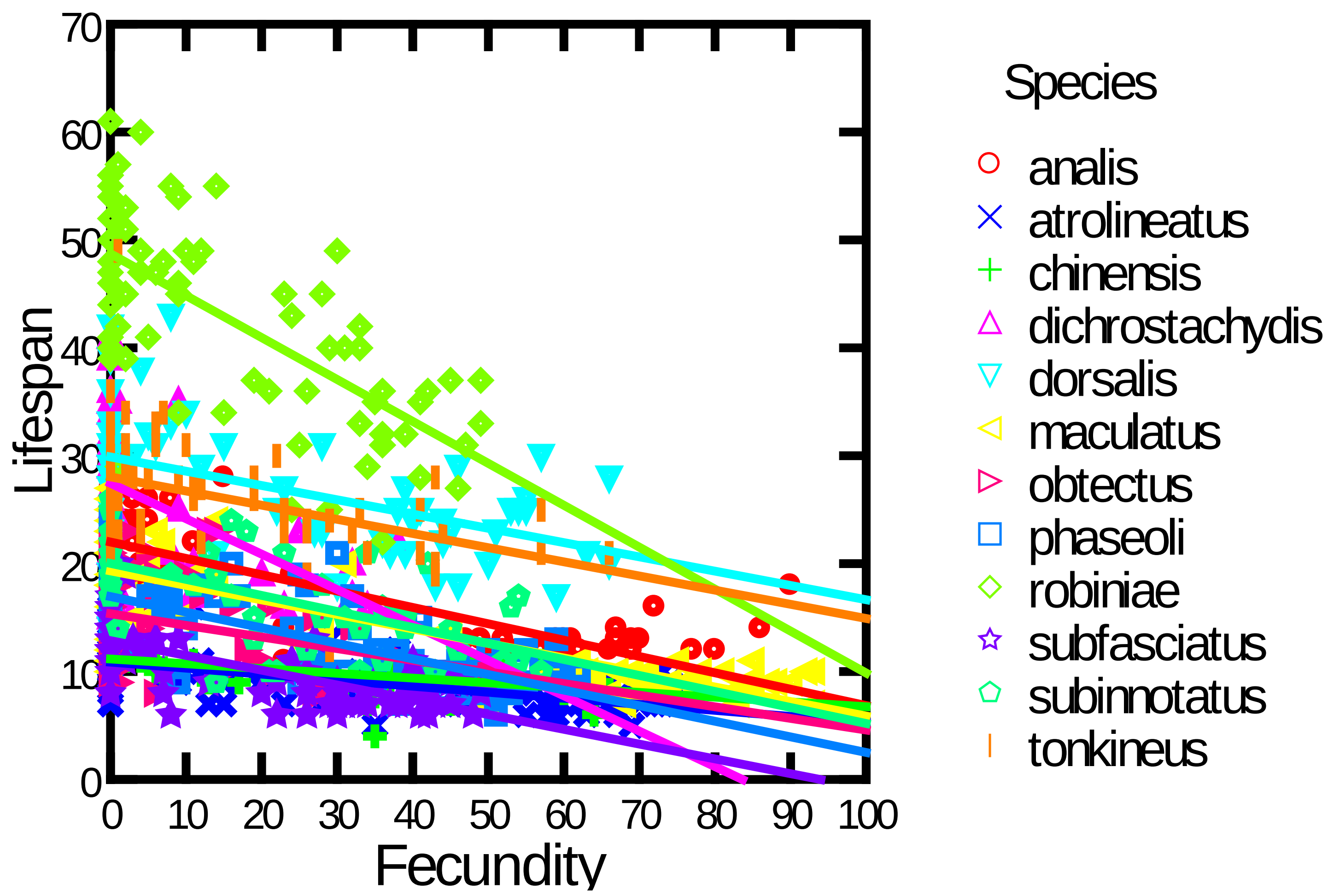

Figure S2. Female lifespan differs markedly between species ($F_{11,814}$ = 239.8, P > 0.001) and female lifespan decreases with the number of eggs laid ($F_{1,814}$ = 353.3, P < 0.001). However, the slope of this relationship differs across species ($F_{11,814}$ = 11.8, P < 0.001). Included here are all data for all females from all four treatment groups in the cost of mating experiment (total N = 843).

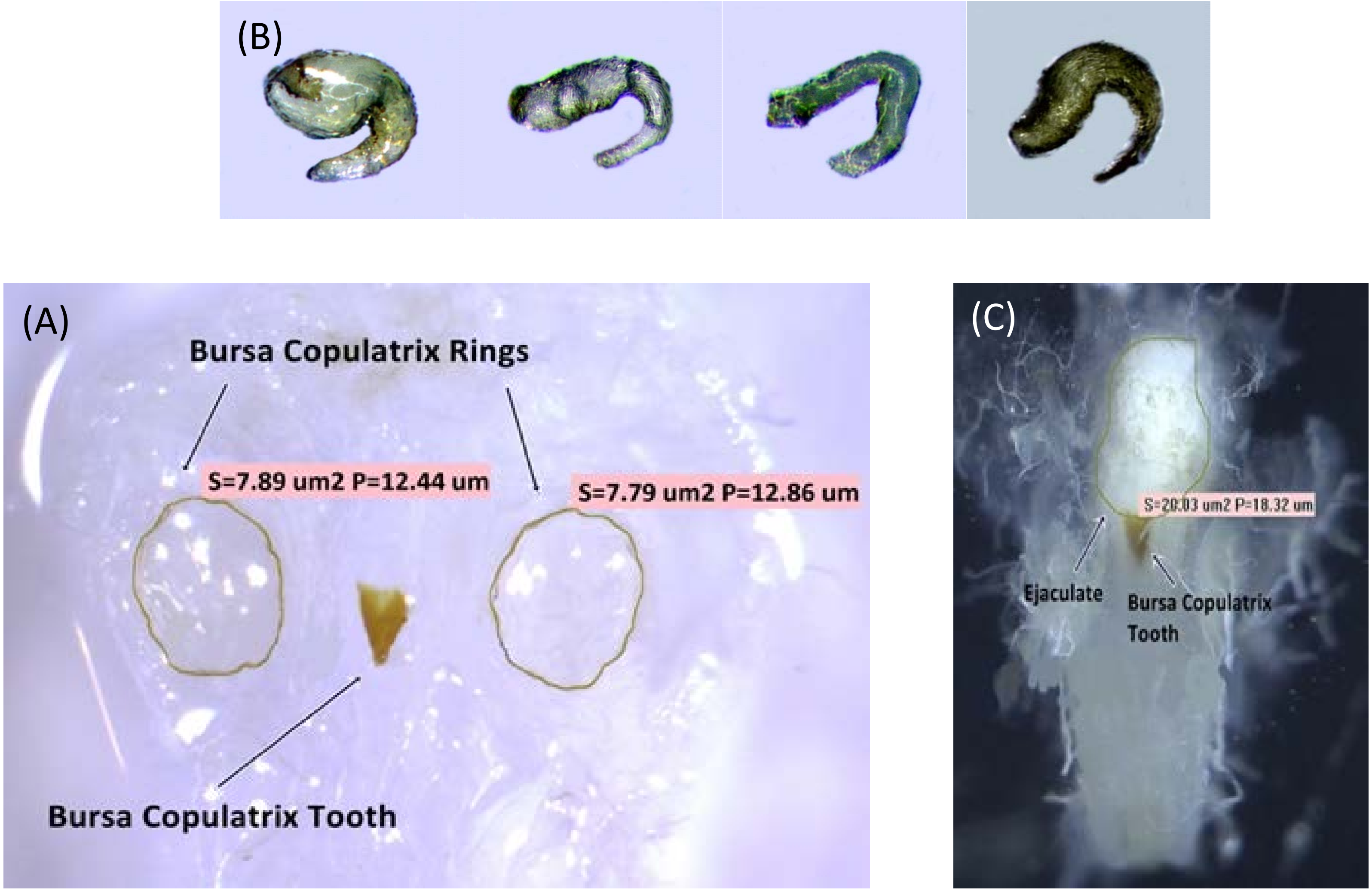

Figure S3. Images illustrating (A) the presence of rings and the ”tooth” in the wall of the bursa copulatrix (*C. subinnotatus*) and (B) variation in the morphology of the spermatheca (left to right: *A. robiniae*, *C. analis*, *Z. subfasciatus* and *M. dorsalis*). Image (C) shows the male ejaculate (encircled) visible through the wall of the bursa copulatrix of a *C. subinnotatus* female, 24 hrs after copulation.

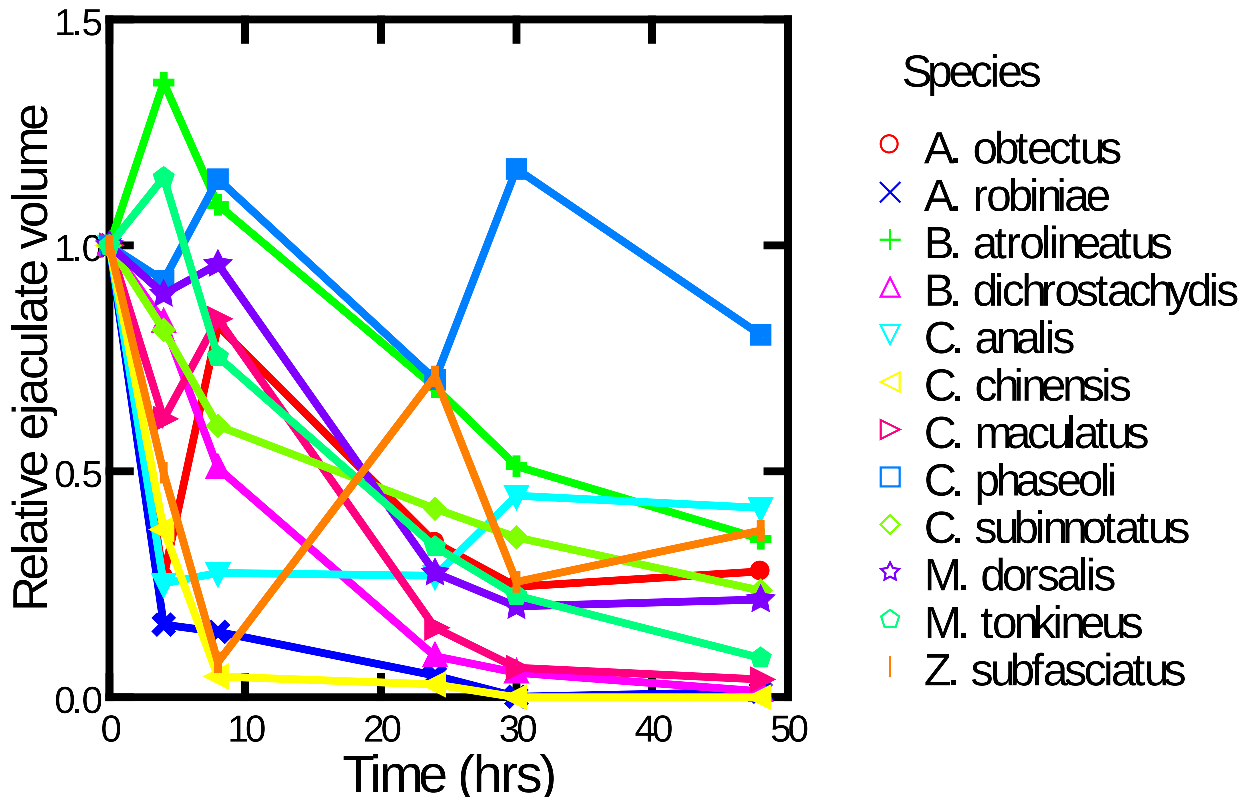

Figure S4. Species differ dramatically in the rate-of-decay of the ejaculate within the female bursa copulatrix (ANCOVA with an intercept = 1; Time × Species: $F_{11,336} = 5.51$, $P < 0.001$). This figure shows the remaining volume of the ejaculate, relative to the average volume at time 0, after 4, 8, 24, 30 and 48 hours after mating. Given here are mean values for each species and time point.

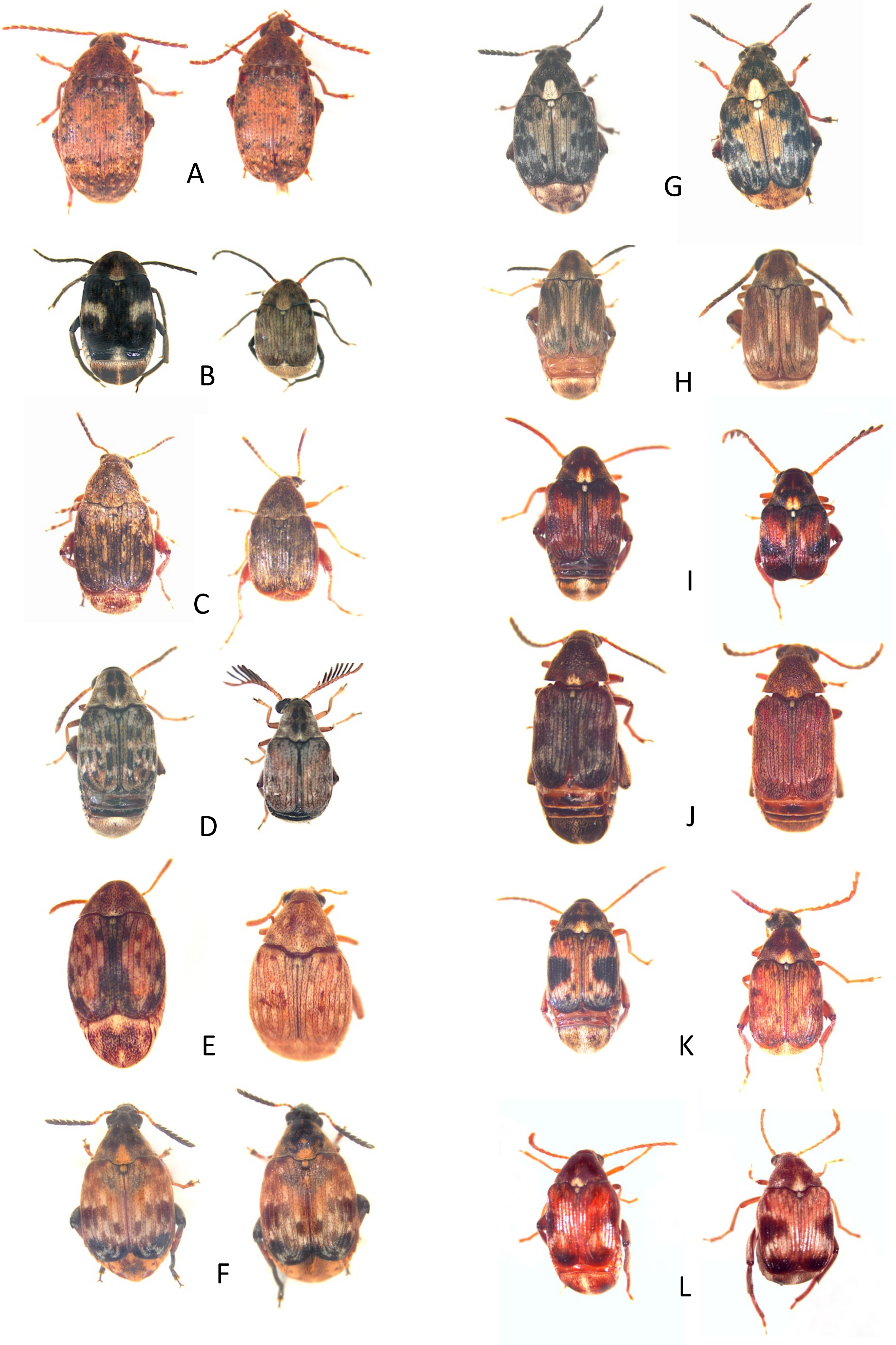
A
B
C
D
E
F
G
H
I
J
K
L

Figure S5. Light microscopy images of all species (females left, males right), to illustrate diversity and sexual dimoprhism: *A. robiniae* (A), *Z. subfasciatus* (B), *A. obtectus* (C), *D. atrolineatus* (D), *B. dichrostachydis* (E), *M. tonkineus* (F), *M. dorsalis* (G), *C. phaseoli* (H), *C. chinensis* (I), *C. subinnotatus* (J), *C. maculatus* (K) and *C. analis* (L). Note that pictures are not to scale.

Table S1. Repeated measures ANOVA of the effects of species, sex and mating status on weight-specific resting metabolic rate.

**Between Subjects Effects**	**SS**	**df**	***F***	***P***
Species	5.73E-02	11	388.4	0.000
Sex	6.79E-05	1	5.1	0.025
Mating status	1.21E-04	1	9.0	0.003
Mating status x Species	8.95E-04	11	6.1	0.000
Sex x Species	4.34E-03	11	29.4	0.000
Mating status x Sex	1.21E-04	1	9.0	0.003
Mating status x Sex x Species	4.95E-04	11	3.4	0.000
Body weight	3.32E-03	1	247.2	0.000
Error	9.38E-03	699		

**Within Subjects Effects**	**SS**	**df**	***F***	***P***	***P (G-G)***	***P (H-F)***
Cycle	4.50E-06	3	1.2	0.327	0.321	0.323
Cycle x Species	9.25E-04	33	21.7	0.000	0.000	0.000
Cycle x Sex	1.32E-04	3	34.0	0.000	0.000	0.000
Cycle x Mating status	3.69E-05	3	9.5	0.000	0.000	0.000
Cycle x Body weight	4.60E-06	3	1.2	0.316	0.311	0.313
Cycle x Mating status x Species	6.11E-05	33	1.4	0.053	0.076	0.070
Cycle x Sex x Species	4.34E-04	33	10.2	0.000	0.000	0.000
Cycle x Mating status x Sex	6.20E-06	3	1.6	0.185	0.196	0.194
Cycle x Mating status x Sex x Species	5.33E-05	33	1.2	0.157	0.184	0.178
Error	2.71E-03	2 097				

Table S2. Repeated measures ANOVA of the effects of species, sex and mating status on respiratory quotient (RQ).

**Between Subjects Effects**	**SS**	**df**	***F***	***P***
Species	0.372	11	6.8	0.000
Sex	0.043	1	8.6	0.004
Mating status	0.023	1	4.6	0.032
Mating status x Species	0.155	11	2.8	0.001
Sex x Species	0.125	11	2.3	0.010
Mating status x Sex	0.000	1	0.1	0.792
Mating status x Sex x Species	0.020	11	0.4	0.967
Body weight	0.016	1	3.2	0.072
Error	3.491	702		

**Within Subjects Effects**	**SS**	**df**	***F***	***P***	***P (G-G)***	***P (H-F)***
Cycle	0.034	3	8.4	0.000	0.000	0.000
Cycle x Species	0.090	33	2.0	0.001	0.001	0.001
Cycle x Sex	0.008	3	2.0	0.115	0.118	0.115
Cycle x Mating status	0.002	3	0.6	0.638	0.630	0.638
Cycle x Body weight	0.029	3	7.3	0.000	0.000	0.000
Cycle x Mating status x Species	0.043	33	1.0	0.530	0.528	0.530
Cycle x Sex x Species	0.076	33	1.7	0.008	0.009	0.008
Cycle x Mating status x Sex	0.002	3	0.6	0.647	0.639	0.647
Cycle x Mating status x Sex x Species	0.042	33	0.9	0.575	0.572	0.575
Error	2.845	2 106				

Table S3. Phylogenetic signal for all 28 traits. Given are Blomberg's K and Pagel's λ, with associated P-values for tests of H_0 : metric = 0.

**Trait**	**K**	***P***	**λ**	***P***
Body weight males	0.62	0.149	1.00	0.066
Body weight females	0.64	0.180	**1.00**	**0.036**
Lifespan females	0.61	0.199	1.00	0.055
Lifespan males	0.66	0.150	0.97	0.154
Fecundity	0.37	0.569	0.05	0.941
PO females	0.60	0.171	0.42	0.439
PO males	0.46	0.359	0.00	1.000
RMR females	**0.87**	**0.031**	**1.00**	**0.024**
RMR males	0.27	0.843	0.00	1.000
RQ females	**0.87**	**0.038**	0.93	0.076
RQ males	**0.94**	**0.021**	**1.00**	**0.036**
Ejaculate volume (um^3)	**0.98**	**0.020**	1.00	0.059
Cost of reproduction females	0.39	0.522	0.00	1.000
Cost of mating females	0.53	0.229	0.00	1.000
Cost of mult mating females LS	0.33	0.637	0.00	1.000
Cost of mult mating females FEC	0.36	0.650	0.00	1.000
Cost of mating males	**0.80**	**0.034**	1.00	0.172
Ejaculate decay rate	0.60	0.200	1.00	0.146
Mating duration	0.38	0.486	0.00	1.000
Bursa area	0.63	0.119	1.00	0.109
Spermatheca area	0.57	0.220	1.00	0.075
Ring prescence	**1.30**	**0.009**	**1.00**	**0.016**
Tooth prescence	**0.96**	**0.031**	1.00	0.068
Genital harmfulness	0.53	0.225	0.00	1.000
RQ change females	0.31	0.671	0.00	1.000
RQ change males	0.40	0.584	0.00	1.000
RMR change females	0.43	0.383	0.00	1.000
RMR change males	0.27	0.824	0.00	1.000

Table S4. Phylogenetic least squares models of trait evolution.

**Response**	**Predictor**	**Coeff.**	**SE**	***t***	***P***
Cost of mating in females	Ejaculate size	-5.35E-04	1.03E-04	-5.22	0.002
	Male genital harmfulness	2.28E-04	8.12E-03	0.03	0.979
	PO activity females	-3.39E-01	5.15E-01	-0.66	0.535
	Bursa size	3.78E-04	1.99E-04	1.90	0.107
	Bursa ring presence	8.38E-02	3.35E-02	2.51	0.046
Cost of multiple mating in females (LS)	Ejaculate size	2.96E-04	2.82E-04	1.05	0.334
	Male genital harmfulness	5.50E-02	2.23E-02	2.46	0.049
	PO activity females	-2.32E+00	1.42E+00	-1.64	0.152
	Bursa size	-1.15E-03	5.49E-04	-2.09	0.082
	Bursa ring presence	8.74E-03	9.20E-02	0.09	0.927
Cost of mating in males	Ejaculate size	2.54E-04	8.95E-05	2.84	0.025
	RMR change males	2.70E-01	4.00E-01	0.68	0.521
	RQ change males	-1.83E+00	1.31E+00	-1.40	0.205
	PO activity males	-6.33E-01	7.69E-01	-0.82	0.438
PO activity females	Male genital harmfulness	1.04E-02	3.20E-03	3.24	0.018
	Lifespan females	1.70E-03	1.21E-03	1.40	0.210
	Fecundity	7.62E-04	4.76E-04	1.60	0.161
	RMR females	1.53E-02	1.97E-02	0.78	0.465
	Body weight females	-2.24E-03	1.16E-03	-1.93	0.102

DATASETS (included as separate files)

Dataset S1. Mean values for all sex-specific traits across species (i.e., tip data).

Dataset S2. Correlation matrix for the 28 species specific measures of life history and economics of mating.

Dataset S3. Correlation matrix for size adjusted species specific measures of life history and economics of mating.

Dataset S4. Phylogenetic correlation matrix for the 28 species specific measures of life history and economics of mating.
